# Whole exome sequencing of microdissected splenic marginal zone lymphoma: a study to discover novel tumor-specific mutations

**DOI:** 10.1186/s12885-015-1766-z

**Published:** 2015-10-24

**Authors:** Jan Peveling-Oberhag, Franziska Wolters, Claudia Döring, Dirk Walter, Ludger Sellmann, René Scholtysik, Marco Lucioni, Max Schubach, Marco Paulli, Saskia Biskup, Stefan Zeuzem, Ralf Küppers, Martin-Leo Hansmann

**Affiliations:** 1 Medizinische Klinik 1, Klinikum der Johann Wolfgang Goethe-Universität, Theodor-Stern-Kai 7, Frankfurt am Main, Germany; 2 Senckenbergisches Institut für Pathologie, Klinikum der Johann Wolfgang Goethe-Universität, Theodor-Stern-Kai 7, Frankfurt am Main, Germany; 3 Institute of Cell Biology (Cancer Research), Medical School, University of Duisburg-Essen, Essen, Germany; 4 Department of Human Pathology, Fondazione IRCCS Policlinico San Matteo, University of Pavia, Pavia, Italy; 5 Institute of Medical Genetics and Human Genetics, Charité Universitätsmedizin Berlin, Augustenburger Platz 1, Berlin, Germany; 6 CeGaT GmbH, Paul-Ehrlich-Straße 23, Tübingen, Germany; 7 German Cancer Consortium (DKTK), Heidelberg, Germany

**Keywords:** Splenic marginal zone lymphoma, Lymphoma, Next generation sequencing, SMYD1, NOTCH2

## Abstract

**Background:**

Splenic marginal zone lymphoma (SMZL) is an indolent B-cell non-Hodgkin lymphoma and represents the most common primary malignancy of the spleen. Its precise molecular pathogenesis is still unknown and specific molecular markers for diagnosis or possible targets for causal therapies are lacking.

**Methods:**

We performed whole exome sequencing (WES) and copy number analysis from laser-microdissected tumor cells of two primary SMZL discovery cases. Selected somatic single nucleotide variants (SNVs) were analyzed using pyrosequencing and Sanger sequencing in an independent validation cohort.

**Results:**

Overall, 25 nonsynonymous somatic SNVs were identified, including known mutations in the *NOTCH2* and *MYD88* genes. Twenty-three of the mutations have not been associated with SMZL before. Many of these seem to be subclonal. Screening of 24 additional SMZL for mutations at the same positions found mutated in the WES approach revealed no recurrence of mutations for *ZNF608* and *PDE10A*, whereas the *MYD88* L265P missense mutation was identified in 15 % of cases. An analysis of the *NOTCH2* PEST domain and the whole coding region of the transcription factor *SMYD1* in eight cases identified no additional case with a *NOTCH2* mutation, but two additional cases with *SMYD1* alterations.

**Conclusions:**

In this first WES approach from microdissected SMZL tissue we confirmed known mutations and discovered new somatic variants. Recurrence of *MYD88* mutations in SMZL was validated, but *NOTCH2* PEST domain mutations were relatively rare (10 % of cases). Recurrent mutations in the transcription factor *SMYD1* have not been described in SMZL before and warrant further investigation.

**Electronic supplementary material:**

The online version of this article (doi:10.1186/s12885-015-1766-z) contains supplementary material, which is available to authorized users.

## Background

Splenic marginal zone lymphoma (SMZL) is a rare low-grade B-cell lymphoma listed as distinct entity in the World Health Organization (WHO) classification of lymphoid neoplasm, accounting for less than 2 % of non-Hodgkin lymphomas [1, 2]. It commonly follows an indolent course exceeding a median 10-year survival. However, in a minority of cases it can pursue a more aggressive course with the possibility of transformation into a diffuse large B-cell lymphoma [3].

The molecular pathogenesis of SMZL is still not entirely clear. There are no signature genetic mutations which allow a reliable clinical diagnosis. Cytogenetic studies in the past identified recurrent deletions of 7q22-q36 and 3q gains in approx. 45–50 % and 20–30 % of cases, respectively [4]. However, within these regions, molecular studies did not identify single genes with an essential role in SMZL pathogenesis [5, 6]. Candidate gene studies, gene expression profiling or miRNA expression studies revealed recurring molecular signatures such as the NF-κB pathway or increased B-cell receptor signaling [7–10]. Several whole-genome sequencing- or whole-exome sequencing (WES) studies identified recurrent somatic mutations involved in the NF-κB pathway [11–14]. Mutations in *NOTCH2*, which eliminate the C-terminal PEST domain and result in compromised protein degradation, were identified in 20–25 % of cases in two studies [11, 14], but such high frequencies were not confirmed in other investigations [12, 13]. Recently, mutations in KLF2, a member of the Krüppel-like family of transcription factors with roles in cell differentiation, proliferation, activation and trafficking were found recurrently mutated in SMZL. First studies showed high frequency of these mutations in up to 40 % of SMZL cases [15, 16], but a consecutive study found lower SNV-frequencies (12 % of cases) [17]. The tumor DNA investigated in the previous studies was retrieved either from whole frozen tissue or paraffine embedded sections or CD19^+^ cells from the peripheral blood or tumor tissue.

In our current study we performed WES from laser-microdissected tumor cells deriving from two cases of SMZL. We confirm several somatic mutations from previous studies and expand the known genetic signature of SMZL by several newly discovered mutations.

## Methods

### Patient selection

Splenic tissue from 26 patients with SMZL was selected for this study from the Department of Pathology, Pavia, Italy, the Department of Pathology, Frankfurt, Germany, and the University of Duisburg-Essen, Medical School, Essen, Germany. Two splenic tissue samples were used to perform next generation sequencing and were selected for their classical tumor morphology, high tumor cell content and availability of fresh tissue (Table 1). For validation experiments, samples of 24 patients were selected who underwent splenectomy for SMZL and featured a tumor cell content of greater than 60 % (estimated by morphology and immunohistochemistry; summary of clinical data presented in Additional file 1: Table S1). Within the validation group, fresh frozen tissue as well as formalin-fixed, paraffin-embedded (FFPE) tissue was available in 8 patients. In the remaining 16 patients, only FFPE tissue was available. The diagnosis of SMZL was established by standard morphological, cytochemical and immunophenotypic methods according to the 2008 WHO lymphoma classification and its diagnostic criteria [2, 18]. All cases included in the study were classical SMZL, with a typical CD5^−^, CD10^−^, Bcl-6^−^, CD23^−^ phenotype and with a typical pattern of white pulp involvement. Patient samples that showed *MYD88* mutations in the current sequencing analysis were re-evaluated to confirm SMZL diagnosis. Informed patient consent was obtained according to the declaration of Helsinki, and the study was ethically approved by the ethics committee of the Medical faculty of the Goethe-University of Frankfurt (Vote #4/09, 2013).

**Table 1:** Clinical characteristics of discovery case 1 and 2

Discovery case	Sex	Age at splenectomy	B-Symptoms	Leukemic disease (villous Lymphozytes)	Immunophenotype	Clinical staging at splenectomy	Oncological response^a^
1	f	59	yes	no	CD20+++, IgM-, Kappa-, Lambda-, Ki-67 5 %, CD5-, CD3-, Cyclin-D1-, bcl-2++	IV	CR
2	f	59	yes	no	CD20+++, IgM+, Kappa-, Lambda-, Ki-67 15 %, CD5-, CD3-, Cyclin-D1-, bcl-2++	IV	CR

^a^after splenectomy

### DNA isolation from tumor cells and non-tumorous controls

For WES, tumor cells were specifically laser-microdissected from fresh frozen tissue sections as described elsewhere [9]. After hemalaun and eosin staining, areas of tumor cells within the splenic marginal zone were selectively microdissected and the tissue was directly transferred into DNA lysis buffer. Representative counting of neoplastic cells within microdissected tissue showed mean tumor cell proportions between 87 and 89 %. The co-analyzed non-tumorous controls were purified from fresh splenic tissue of the corresponding patient using MACS-sorting for CD3^+^ cells. For validation, DNA was isolated from whole tissue slides. DNA purification was performed using the QIAamp DNA extraction kit (QIAGEN, Hilden, Germany).

### Whole exome enrichment and sequencing

Purified tumor and germline genomic DNA (3 μg) from the two discovery SMZL cases was enriched in protein coding sequences using the in-solution exome capture SureSelect Human All Exon 50 Mb kit (Agilent Technologies, Böblingen, Germany), according to the manufacturer’s protocol. WES was performed using the SOLiD4 Platform (Life Technologies, Darmstadt, Germany). Each sample was sequenced on a single quad of a SOLiD sequencing slide (Life Technologies).

### Single nucleotide polymorphism (SNP)-array analysis

Tumor and germ-line DNA was purified, amplified, labelled and hybridized to the Affymetrix SNP5.0 platform (Affymetrix, Santa Clara, CA) according to the manufacturer’s protocol. Copy number profiles (aligned to hg19/GRCh37) from tumor and germ-line samples were compared using the Genotyping Console software (Affymetrix). The minimum number of SNP-markers per segment were set to 5 with a minimum genomic size of a segment of 100 kb. Allele ratio and copy number neutral loss of heterozygosity were calculated for each sample using the HapMap Allele Reference baseline (Affymetrix).

### Validation with pyrosequencing and Sanger sequencing

The pyrosequencing technique was used for validation of specific discovered single base substitution-type mutations (*NOTCH2* C7310T, *SMYD1* G839T, *MYD88* T794C, *ZNF608* A3659G, *PDE10A* G1072A) in a validation cohort of 24 microdissected SMZL samples. An internal fragment of each gene was amplified by polymerase chain reaction (PCR) using primers specific for each gene and a PyroMark PCR kit (QIAGEN). The resulting PCR products were sequenced with the PyroMark Q24 (QIAGEN) pyrosequencer using PyroMark Gold Q96 reagents (QIAGEN) and sequencing primers specific for each gene.

Conventional Sanger sequencing was used to screen for mutations in two selected genes within the validation cohort (*NOTCH2* and *SMYD1*). The PEST domain of *NOTCH2* within exon 34 and all 10 exons of *SMYD* were sequenced in 8 validation and 2 WES cases. Primer sequences are given in Additional file 2: Table S2; Additional file 3: Table S3; Additional file 4: Table S4.

### Bioinformatics and statistical analyses

Next generation sequencing reads were mapped with the Blast-like mapping algorithm from Bioscope v1.2 (Life Technologies) against the human reference genome (hg19 from UCSC) using color codes. High sensitive variant calling-including small insertions and deletions as well as single nucleotide variants (SNVs)-was performed by the DiBayes algorithm from Bioscope. Transcript and protein alterations were annotated with NGS-SNP [19] using the ENSEMBL v61 database [20, 21]. Only variants potentially changing the protein sequence were used for further analysis; intronic, UTR and synonymous mutations were removed. In addition, low quality SNVs were filtered out using the novel allele mean quality given by Bioscope. All SNVs that were below the mean minus two times the standard deviation from a calling were discarded.

To find somatic variants, only SNVs with a minimum coverage of 20 and a minimum novel allele frequency of 0.1 were intersected with the variants from the normal tissue. Somatic variants were called if the allele frequency of the normal tissue was smaller 0.2 and the delta between tumor and normal frequency was at least 0.1. All SNVs identified by this algorithm underwent manual review by two independent observers using the integrative genomics viewer (Broad Institute, v1.5) to reduce the rate of false positives [22]. Protein illustration was performed using ballView software (http://www.ballview.org/).

## Results

**Fig. 1:**
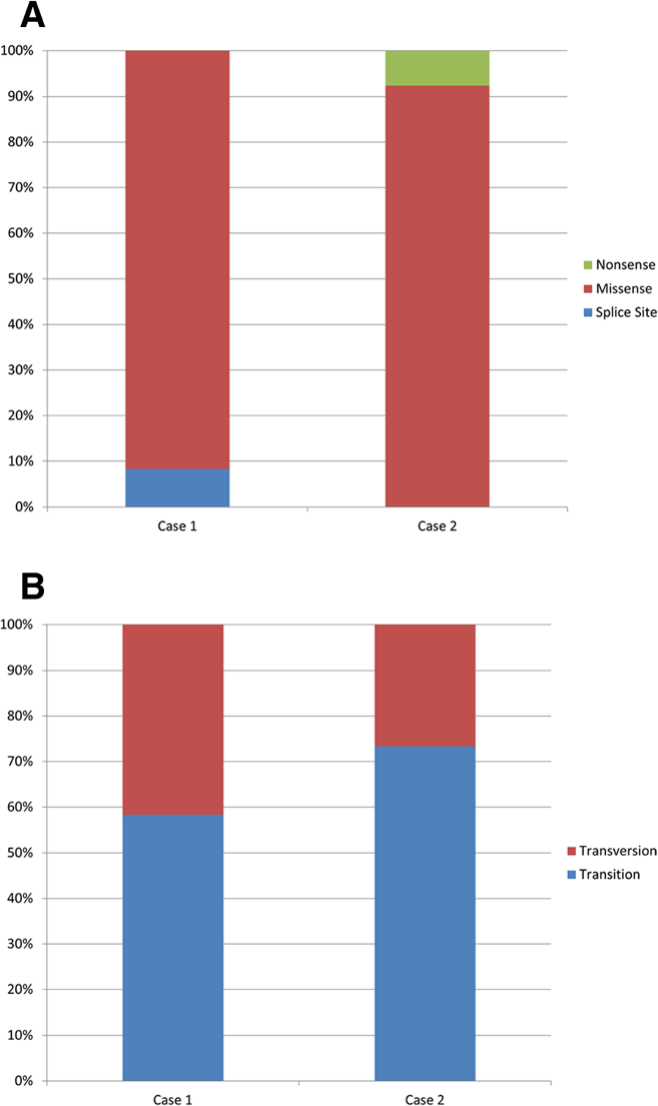
Distribution of single nucleotide variants in the coding SMZL genome. **a**. Relative pattern of somatic mutations identified in two SMZL discovery cases using WES. **b**. Relative distribution of transitions and transversions in discovery cases 1 and 2

Two well-matched SMZL patients (both female, same age at splenectomy, similar morphology and immunophenotype of SMZL; Table 1) were chosen for NGS analysis. Exome-capture and high-throughput sequencing of microdissected lymphoma cells from two SMZL allowed us to align approx. 62.1 million reads per sample with an off-target read number of 3.4 million (5.5 %) at a mean depth of 47-fold (range 42–49). In total, an average of 77, 66 and 54 % of target sequences were captured at a minimum coverage of 10, 20 and 30, respectively. Our analytical algorithm identified 216 non-synonymous variants (Additional file 5: Table S5). Of these, 191 were filtered out, as they were either identified as previously described polymorphisms or showed high incidence in the non-tumorous matched controls, leaving 25 probable somatic mutations, 12 in case 1 and 13 in case 2 (Table 2). Of the 25 base substitutions, 23 were missense mutations, one was a splice site, and one was a nonsense mutation (Fig. 1a). In addition 64 and 36 % of these variants were transitions and transversions, respectively (Fig. 1b).

**Table 2:** Somatic mutations in the two SMZL analyzed by WES

Gene	Case	Chr	Position	Exon	Coverage tumor	SNV frequency	Quality score^a^	Nucleotide change	AA change	SIFT prediction	PolyPhen-2 prediction
CEBPZ	1	2	37.456.150	2	21	0.38	27.0	c.186 T > A	p.D62E	Tolerated	Benign
MYD88	1	3	38.182.641	5	49	0.49	30.0	c.794 T > C	p.L265P	Damaging	Probably damaging
TTC14	1	3	180.320.802	2	21	0.24	32.0	c.285A > G	p.E95E^b^	Tolerated	Benign
SLC6A7	1	5	149.574.417	2	33	0.27	23.0	c.160 T > C	p.C54R	Damaging	Probably damaging
BTN2A2	1	6	26.384.057	2	29	0.24	32.0	c.8C > T	p.P3L	Damaging	Possibly damaging
POM121	1	7	72.396.892	5	41	0.29	20.0	c.737C > T	p.P246L	Damaging	Probably damaging
MUC12	1	7	100.645.822	2	32	0.25	31.0	c.12407C > A	p.P4136H	Tolerated	Probably damaging
STMN4	1	8	27.098.767	5	40	0.25	30.0	c.203A > C	p.D68A	Damaging	Benign
KRTAP5-2	1	11	1.619.441	1	20	0.35	27.0	c.40 T > C	p.C14R	N/A	Possibly damaging
CACNA1C	1	12	2.794.937	47	63	0.25	20.0	c.5823C > T	p.T1941M	Tolerated	N/A
LOC728888	1	16	29.395.118		61	0.15	18.0	c.1135G > T	p.G379W	Damaging	Probably damaging
CDC27	1	17	45.247.333	4	42	0.26	28.0	c.327G > T	p.E109D	Tolerated	Benign
FBXO44	2	1	11.718.850	4	21	0.19	20.28	c.421G > A	p.V141I	Tolerated	Benign
NOTCH2	2	1	120.458.255	34	65	0.48	22.0	c.7090C > T	p.Q2364^c^	Stop gained	N/A
SMYD1	2	2	88.396.251	6	26	0.50	26.0	c.836G > T	p.C279F	Damaging	Probably damaging
ZNF608	2	5	123.982.418	4	73	0.33	26.0	c.3659A > G	p.D1220G	Damaging	Probably damaging
ZNF451	2	6	57.013.380	10	30	0.20	24.93	c.2497 T > C	p.F833L	Tolerated	Benign
PDE10A	2	6	165.829.696	13	116	0.37	22.0	c.1072G > A	p.A358T	Tolerated	Probably damaging
PCLO	2	7	82.784.519	2	57	0.18	17.0	c.1438C > T	p.P480S	Tolerated	Benign
PRSS1	2	7	142.460.369	4	26	0.23	19.0	c.542G > A	p.S181N	Tolerated	Benign
CSMD1	2	8	2.800.055	69	74	0.36	24.0	c.8683A > T	p.I2895F	Damaging	N/A
APOA4	2	11	116.692.500	3	20	0.35	31.0	c.274G > A	p.A92T	Tolerated	Benign
HERC2	2	15	28.458.971	42	23	0.22	25.97	c.6703C > A	p.R2235S	Tolerated	Possibly damaging
CDC27	2	17	45.229.185	9	90	0.23	29.0	c.1075G > C	p.G359R	Tolerated	Possibly damaging
GRIN2C	2	17	72.842.984	10	39	0.44	23.0	c.2077C > T	p.R693C	Damaging	Probably damaging

*AA* amino acid, *Chr* chromosome, *N/A* not applicable, *SIFT* Sorting Tolerant from Intolerant Algorithm, *SNV[space]*single nucleotide variant

^a^calculated by Bioscope algorithm

^b^splice site

^c^Stop gained

**Fig. 2:**
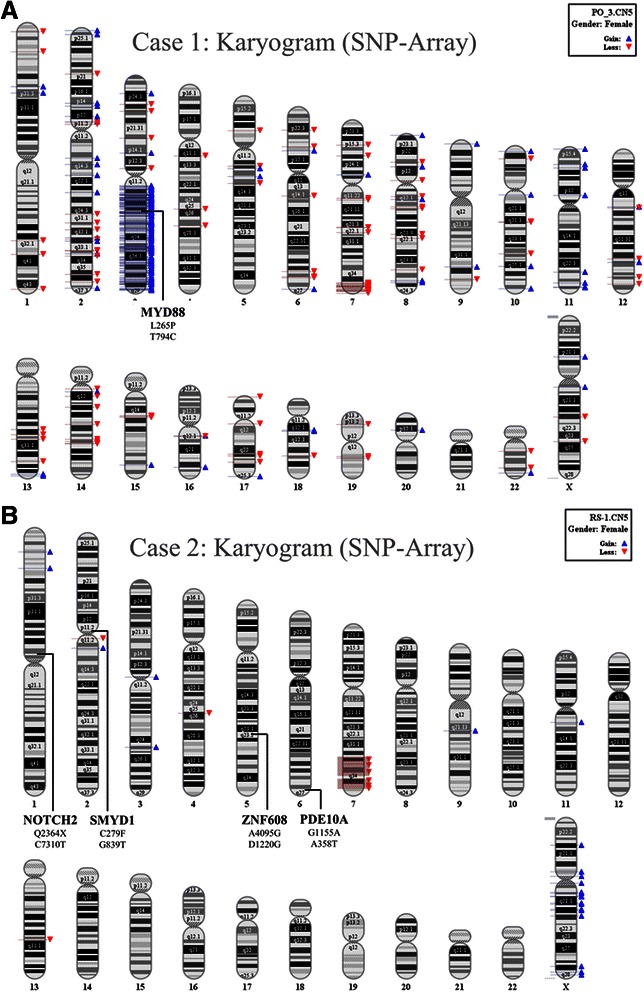
Digital karyogram of SMZL cases. Copy number alterations of the two discovery cases using Affymetrix SNP5.0 platform (minimum number of SNP-markers per segment were set to 5 with a minimum genomic size of a segment of 100 kb). Gains are shown in blue arrows, losses are shown in red arrows. **a**. Discovery case 1. **b**. Discovery case 2

SNP-array analysis of the two SMZL identified 3 somatically-acquired copy number aberrations. Case 2 features a duplication of 3q and both cases harbour a deletion in 7q (Fig. 2), which in both cases covers the 7q32 region previously described in SMZL [4, 6]. Only one of the mutations identified (*MYD88* L265P) falls within a copy number aberration, namely the 3q gain. The *MYD88* L265P mutation in the discovery case with the 3q duplication was called in only one third of the reads (14 of 50) by the WES analysis, so likely the mutation is located on the non-duplicated region of chromosome 3.

Somatic mutations identified in the discovery cases are shown in Table 2. Only two of these (*NOTCH2* and *MYD88*) have been described in SMZL previously [11–14, 23–25]. Twenty-three variants discovered in the current investigation have, to our knowledge, not been described in SMZL before. None of these mutations was found in both cases. Furthermore, none of the somatic mutations showed features of homozygosity (70–100 % frequency of the mutated variant) and only 9 mutations (36 %) showed a minor allele call of >35 %. SIFT algorithm predicted about half of the mutations to be damaging (10/25 variants damaging, 1/25 stop gained, Table 2) [26]. Similar predictions were made by the Polyphen-2 algorithm [27], which classified 13/25 variants to be possibly or probably damaging (Table 2). As mutations in *KLF2* have been found with high frequencies in SMZL in other studies, this region was specifically reviewed within the current WES data. The bioinformatic SNP-detection algorithm did not detect any mutations in this area. Also manual review of the region of interest did not detect KLF2 mutations. However, large areas of this gene showed poor coverage (mean coverage, range: 12.6, 1–44), although the enrichment design included specific baits for this region. The coverage statistics of other selected genes of interest mentioned in this study are shown in Additional file 6: Table S6.

Five of the 25 genes were chosen for experimental validation of the variants identified in the WES study in an independent cohort. We chose *NOTCH2* and *MYD88* for their known recurrence in SMZL and the genes *SMYD1*, *PDE10A*, and *ZNF608*, which had not been previously described to be mutated in this tumor entity. Pyrosequencing confirmed the somatic origin of these five mutations in the respective discovery case. Moreover, we screened a validation cohort consisting of 24 SMZL FFPE samples for mutations. Pyrosequencing of the same base position previously found mutated in the discovery cohort was used for technical reasons, as quality of FFPE tissue was insufficient for sequencing of the complete coding regions of the genes. Pyrosequencing showed no recurrent variants at *NOTCH2* C7310T, *SMYD1* G839T, *ZNF608* A3659G, *PDE10A* G1072A while *MYD88* T794C was found in 3 out of 24 validation cases (12.5 %, Additional file 7: Figure S1).

**Fig. 3:**
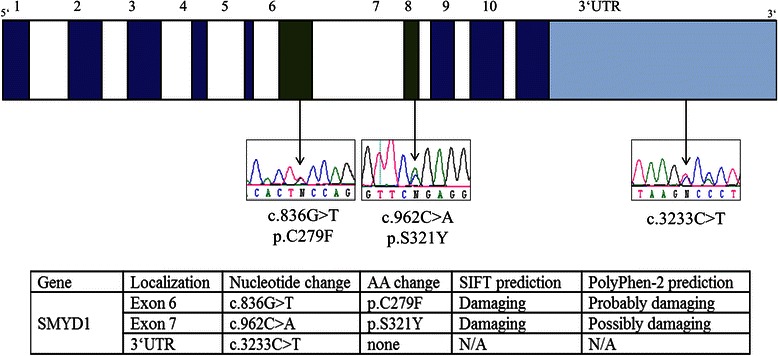
Sanger sequencing of SMYD1. All exons of the *SMYD1* gene were sequenced using conventional Sanger sequencing. Only forward sequences are shown, reverse sequencing showed corresponding results. Schematic illustration of the gene and the mutations identified. Exons with mutations are depicted in green and those without mutations in blue. SIFT and PolyPhen-2 predictions for *SMYD1* mutations are shown in the table

Sanger sequencing was used to screen all exons of SMYD1 and the complete PEST domain of *NOTCH2* for mutations in those eight patients of the validation cohort where fresh tissue was available. While Sanger sequencing was able to confirm the SNV detected in *NOTCH2* in the discovery case itself, no additional mutation within the PEST domain of *NOTCH2* was found within the validation cohort (Additional file 8: Figure S2). Including the discovery case, three mutations were detected in *SMYD1* (30 % of screened cases) localized in exons 6, 7 and the 3’ untranslated region (UTR) (Fig. 3). Sanger sequencing of non-tumorous germline DNA of each patient was used to ensure the somatic origin of the SMYD1 mutations. We compared our findings to three NGS databases: 1000 genome project [28], Exome Aggregation Consortium database (ExAC, Cambridge, MA [http://exac.broadinstitute.org]), Catalogue of somatic mutations in cancer (COSMIC) [29]. These databases incorporate over 60,000 sequenced exomes with partly overlapping content. The SMYD1 mutations found in our current SMZL cohort are not described in one of the mentioned platforms. SMYD1 seems rather well conserved with only 189 missense or loss-of-funcion variants throughout the gene. COSMIC database contains 170 unique cancer samples with SMYD1 mutations out of 24,615 total samples. Mutations have been discovered in various cancer types (e.g., gastric-, hepatocellular-, bladder-, renal cell carcinoma, melanoma or glioma).

Both exonic mutations are predicted to be damaging by SIFT and Polyphen-2 algorithm and are located within the catalytic SET domain of SMYD1. Moreover, an analysis using Universal Protein Resource (UniProt) by the European Bioinformatics Institute [30] shows that one of the found mutations (c.836G ≥ T; p.C279F) is located at one of the four zinc binding sites of the catalytic centre of SMYD1. Furthermore, we used the crystal structure of the murine SMYD1 protein (retrieved from the Protein Data Bank; PDB-ID: 3 N71 [30, 31]) and localized the detected SMYD1 mutations (Additional file 9: Figure S3). The p.S321Y mutation lies in an exposed position within the functional pocket of the protein. Moreover, the region of the 3’ UTR where the SNV was found is predicted to be a high fidelity target of miR-28 (seed length 11, *p* = 0.0007) by the miRWalk platform [32].

## Discussion

This work is the first of its nature to study the whole exome of microdissected SMZL cells. It demonstrates the feasibility of this approach and largely confirms the sequencing results of previous exome sequencing approaches. However, novel mutations in SMZL were discovered, expanding the directory of reported variants. It has to be acknowledged that through bioinformatics selection of putative SNVs using coverage and allele-frequency criteria, significant amounts of mutations might have been lost while falsely positive SNPs may still have been detected. Manual review of all called SNVs was used to avoid such errors.

### Clonality of mutations in SMZL

The number of SMZL analyzed by WES in the current study is small, but laser-microdissection as the method of tumor cell isolation ensures a high purity of tumor DNA for the sequencing. WES with the currently available number of sequencing reads per NGS run has clear limitations in sequencing coverage. Therefore, it is not an ideal method to detect subclonal mutations, especially compared to ultra-deep targeted re-sequencing approaches. Nevertheless, high tumor cell purity may enable also WES to screen for potential subclonal aberrations. We identified mutations that seem to be heterozygous and clonal as they show allele frequencies of >40 % in the WES and 46–53 % in the validation experiments (*NOTCH2*, *SMYD1*, *MYD88*), with the proximity to 50 % providing an indirect evidence for high tumor cell purity. However, 12 out of 25 somatic mutations showed mutation frequencies below or equal to 25 %, leaving a majority of SNVs likely subclonal variants. This might be a main reason why these genes have not been found as mutated in prior sequencing studies. Although the finding of many presumably subclonal mutations remains to be validated in future in depth WES studies with isolated lymphoma cells, we provide here intial evidence for a remarkable intraclonal diversification during SMZL clonal expansion.

### Mutations in NOTCH2 and MYD88

Of the 25 non-synonymous variants found within the two discovery cases of the current analysis, *NOTCH2* and *MYD88* were already known to be recurrently mutated in SMZL [11–14, 23–25]. The heterodimeric transmembrane protein *NOTCH2* has been found mutated in 21–25 % of SMZL by the pivotal studies [11, 14], but in lower frequency in others [12, 13]. *NOTCH2* plays an important role in marginal zone B cell development in the spleen and mutations in its pathway were identified in various B-cell lymphomas [33–35]. In our cohort, although present in one of the discovery cases, we did not detect further *NOTCH2* mutations at the same base position in the validation cases by pyrosequencing (1/24 cases, 4.2 %). As loss-of-function variants, as in *NOTCH2*, rarely concur at the same base position we sequenced also the functional PEST-domain, still discovering only one variant in ten cases. Discrepancies might be explained by technical issues, e.g., use of FFPE material in studies with lower *NOTCH2* mutation frequency. Another explanation could be geographic differences in SMZL etiology, as infectious components like chronic hepatitis C virus infection are thought to be involved [36].

The cytoplasmatic adaptor *MYD88* mediating toll-like receptor induced NF-κB activation has been frequently found mutated in SMZL (10–15 %), in targeted sequencing approaches as well as WES [10, 12, 24, 37]. Especially the L265P missense substitution occurs with high prevalence in various B-cell malignancies [38]. We discovered the L265P variant of *MYD88* in 1 of 2 discovery cases and 3 of 24 validation cases in our cohort. The mutation frequency of 15 % is therefore similar to published data.

The transcription factor KLF2, has been found recurrently mutated in SMZL in recent sequencing studies [15–17]. We did not detect any KLF2 mutations. However, large areas of this gene showed poor sequencing coverage, likely due to the high GC content of KLF2. Also the ExAC database shows that throughout 60,706 incorporated exomes there is largely no or minimal coverage of KLF2. Therefore, we might have failed to detect actual KLF2 mutations in our cases.

### Novel mutations in SMZL

It has to be acknowledged that a WES approach as used in the current study can generate a certain statistical amount of false positive findings [39]. We therefore tried to use manual review of mutation sites and multiple online databases as well as literature review to consolidate our findings. Some of the genes bearing mutations in the SMZL cases investigated here, including *PRSS1* [40], *PCLO* [41, 42], *CSMD1* [26, 43], *HERC2* [44], and *MUC12* [45] have been previously described to be mutated in other tumor entities, but not in SMZL. We identified one gene, SMYD1, with recurrent somatic mutations in our collection of SMZL, which has not been specifically associated to cancer in the past. However, other members of the SMYD family have putative oncogenic roles. The *SMYD* family (*SMYD1*-*5*) is a group of SET domain-containing transcriptional regulators acting mainly through histone modification [46]. *SMYD2* has oncogenic properties by repressing the activity of p53 and RB through methylation [47, 48] and *SMYD3* is commonly upregulated in hepatocellular carcinoma, colon carcinoma and breast cancer with influence on cell proliferation in vitro [49–52]. *SMYD1*, which we found mutated in 3 of 10 SMZL cases, has been associated with cell differentiation and embryogenesis of the heart [53]. It acts through the lysine methyltransferase activity of the SET domain, regulating the muscle-specific transcription factor *skNAC* and is itself targeted by *MEF2C* during cardiac morphogenesis [54–56]. The two coding mutations found in SMYD1 are both located within this SET domain. The p.C279F mutations is located at one of the four zinc binding sites of the catalytic centre of SMYD1. Furthermore, the p.S321Y mutation projects to the functional pocket of the murine crystal structure of the protein. Interestingly, *SMYD1* seems to be involved in *NOTCH1* mediated signalling, which could explain a possible involvement in SMZL oncogenesis [57]. Moreover, the position in the 3’ UTR of *SMYD1*, where we found one of the mutations, is a predicted target of miR28, a recently described potent tumor suppressor in B-cell lymphomagenesis [58].

## Conclusions

In this small study, we performed WES and copy number analysis from microdissected SMZL cells. It demonstrates the feasibility of this approach and demonstrates advantages of analysing high tumor cell purity even in times of ultra-deep sequencing. While some mutations found in previous studies could be confirmed, we also expand the reported directory of mutated genes in this lymphoma entity. *SMYD1*, a gene that has been primarily linked to differentiation and embryogenesis of the heart, was recurrently mutated in our cohort. Similar to closely related genes of the same family (*SMYD2*, *SMYD3*), it might play a role in the oncogenesis of SMZL as member of the *NOTCH1* signalling pathway. Further functional investigation and studies in larger cohorts will be needed to substantiate this hypothesis.

## Additional files

Additional file 1: Table S1. Clinical characteristics of all SMZL cases. (DOC 29 kb)
Additional file 2: Table S2. Primers for PCR and pyrosequencing for SNV validation. (DOC 35 kb)
Additional file 3: Table S3. Primers for Sanger sequencing of the NOTCH2 PEST domain. (DOC 32 kb)
Additional file 4: Table S4. Primers for Sanger sequencing of SMYD1 all exons. (DOC 50 kb)
Additional file 5: Table S5. Characteristics of the identified 216 non-synonymous variants. (XLSX 51 kb)
Additional file 6: Table S6. Coverage statistics for genes of interest. (DOC 30 kb)
Additional file 7: Figure S1. Pyrosequencing of validation cohort. Five genes with SNVs in the discovery cohort were analysed for recurrent mutations in a validation cohort (*n* = 24). Only the SNV position of the respective discovery case was analysed. A. *MYD88* 794 T > C. B. *NOCTH2* 7090C > T. C. *SMYD1* 836G > T. D. *PDE10A* 1072G > A. E. *ZNF608* 3659A > G. (DOC 291 kb)
Additional file 8: Figure S2. Sanger validation of *NOTCH2* p.Q2364* nonsense mutation. Nucleotide substitution c.7090C > T in the *NOTCH2* PEST domain within exon 34. (DOC 263 kb)
Additional file 9: Figure S3. Crystalline structure of the murine SMYD1. Coding mutations found in SMZL are highlighted (c.836G > T; p.C279F in blue, c.962C > A; p.S321Y in red). (DOC 232 kb)

## Abbreviations

FFPE: Formalin-fixed, paraffin-embedded
SMZL: Splenic marginal zone lymphoma
SNP: Single nucleotide polymorphism
SNVs: Single nucleotide variants
UTR: Untranslated region
WES: Whole-exome sequencing
