# Supplementary material for: Whole exome sequencing of microdissected splenic marginal zone lymphoma: a study to discover novel tumor-specific mutations
Source: BMC Cancer. 2015 Oct 24;15:773. doi: 10.1186/s12885-015-1766-z (PMC4619476; doi:10.1186/s12885-015-1766-z)
Supplement: Additional file 1: Table S1. — Clinical characteristics of all SMZL cases. (DOC 29 kb) [file 12885_2015_1766_MOESM1_ESM.doc]

**Table S1** Clinical characteristics of all SMZL cases

| Total | 26 |
| --- | --- |
| Sex (m:f) | 8:18 |
| Average age at splenectomy (range) | 60 (37-78) |
| B-Symptoms (%) | 12 (46) |
| Leukemic disease, villous lymphocytes >10% (%) | 5 (19) |
| Stage IV at splenectomy (%) | 26 (100) |
| Oncological response after splenectomy CR/PR/PD/SD/NA | 10/10/3/1/2 |
| Chemotherapy before Splenectomy (%) | 9 (35) |
