# Supplementary material for: Whole exome sequencing of microdissected splenic marginal zone lymphoma: a study to discover novel tumor-specific mutations
Source: BMC Cancer. 2015 Oct 24;15:773. doi: 10.1186/s12885-015-1766-z (PMC4619476; doi:10.1186/s12885-015-1766-z)
Supplement: Additional file 2: Table S2. — Primers for PCR and pyrosequencing for SNV validation. (DOC 35 kb) [file 12885_2015_1766_MOESM2_ESM.doc]

**Table S2 Primers for PCR and pyrosequencing for SNV validation**

| Gene | Exon | Primer | Sequence (5´-3´) |
| --- | --- | --- | --- |
| NOTCH2 | 34 | Forward | *CTGGCCACCATGTACCAGATT |
|  |  | Reverse | AAGGATGATAGGCTGGGAGAATG |
|  |  | Sequencing | GGAGAATGGTCTGAGC |
| MYD88 | 5 | Forward | *AGGTGCCCATCAGAAGCGA |
|  |  | Reverse | GCTGGGGAACTCTTTCTTCATTG |
|  |  | Sequencing | TTGTACTTGATGGGGAT |
| SMYD1 | 6 | Forward | *AAGAGGCAGCTGAAGAAGCAGTA |
|  |  | Reverse | GTTGTCTTTCACCCCCAGGA |
|  |  | Sequencing | CCTTCAGTTTTTTCTGG |
| ZNF608 | 4 | Forward | *GGAGGCTGTAGAAATGAAGTCTGT |
|  |  | Reverse | GGGTCTACACCTGTCTGCTTCATA |
|  |  | Sequencing | ACACCTGTCTGCTTCATA |
| PDE10A | 13 | Forward | AAGTAGCAAGAACAGGGGAAGTC |
|  |  | Reverse | *GTGGGACATCACAGAAGAAATCA |
|  |  | Sequencing | GAACATTCCAGATGCC |

*biotinylated primers for pyrosequencing
