# Supplementary material for: Whole exome sequencing of microdissected splenic marginal zone lymphoma: a study to discover novel tumor-specific mutations
Source: BMC Cancer. 2015 Oct 24;15:773. doi: 10.1186/s12885-015-1766-z (PMC4619476; doi:10.1186/s12885-015-1766-z)
Supplement: Additional file 3: Table S3. — Primers for Sanger sequencing of the NOTCH2 PEST domain. (DOC 32 kb) [file 12885_2015_1766_MOESM3_ESM.doc]

**Table S3** Primers for Sanger sequencing of the NOTCH2 PEST domain

| Gene | Exon | Primer | Sequence (5`-3`) |
| --- | --- | --- | --- |
| NOTCH2 | Exon 34 Part 1 | Forward | TCCCCTGTTGATTCCCTA |
|  |  | Reverse | CACAATGTGGTGGTGGGATA |
|  | Exon34  Part 2 | Forward | GCACTGTGCTTCCCTCAGT |
|  |  | Reverse | CTGCCTTTAGGGATGAGCTG |
|  | Exon 34  Part 3 | Forward | ACCCATCCTGGCATAGCTC |
|  |  | Reverse | GGTGATGAACTTGACCACTG |
|  | Exon 34  Part 4 | Forward | ACACCCAGTCACAGTGGTCA |
|  |  | Reverse | TGTCTCTACACTGGAGGTGGAC |
