# Supplementary material for: Whole exome sequencing of microdissected splenic marginal zone lymphoma: a study to discover novel tumor-specific mutations
Source: BMC Cancer. 2015 Oct 24;15:773. doi: 10.1186/s12885-015-1766-z (PMC4619476; doi:10.1186/s12885-015-1766-z)
Supplement: Additional file 4: Table S4. — Primers for Sanger sequencing of SMYD1 all exons. (DOC 50 kb) [file 12885_2015_1766_MOESM4_ESM.doc]

**Table S4 Primers for Sanger sequencing of SMYD1 all exons**

| Gene | Location | Primer | Sequence (5’ to 3’) |
| --- | --- | --- | --- |
| SMYD1 | Exon 1 | Forward | CCACCTCCAGGAAAAGTAGCA |
|  |  | Reverse | TGGCAATGATCCCTTGCAGAA |
|  | Exon 2 | Forward | TTCCACTGTGCTCCCAATCAT |
|  |  | Reverse | TTCCTGACTTGCACTCAGCTT |
|  | Exon 3 | Forward | TGCTTGTTTACTTACCTCTCCCC |
|  |  | Reverse | CAGGGCTTCTGCCTCTTAGTT |
|  | Exon 4 | Forward | TCCCTAAGCATCTCCAGGGT |
|  |  | Reverse | TTCTGGAATGAGATTTCTATGTGC |
|  | Exon 5 | Forward | ACCCTTGCACTGGATCACAC |
|  |  | Reverse | GAAGTCTCGGGAATGGGTGG |
|  | Exon 6 | Forward | TGTGGGGTCAATGGGAGTTT |
|  |  | Reverse | GGACCTAGCTGGGACCTACC |
|  | Exon 7 | Forward | TTACAGGAATGAGGCATTCAGA |
|  |  | Reverse | TCATGATCTCAACCAAGGGACA |
|  | Exon 8 | Forward | GTGCTTTCCCACCTCTGTCA |
|  |  | Reverse | AGATGTGCTGGAGAATTTGGGA |
|  | Exon 9 | Forward | GTGCTGAGGGGAAGACAGAT |
|  |  | Reverse | AGTGGGCTAAAGCGTTTCTGA |
|  | Exon 10 | Forward | GAGTTGAATCTCCGTGGCTGG |
|  |  | Reverse | TCACACACCAGACACATTCCC |
|  | 3‘ UTR | Forward | TGGGTGGGGAAGCAAAATGT |
|  |  | Reverse | AATTCTGCAGGGCCCATGAT |
|  | 3‘ UTR | Forward | TTCTGGCTATTTGCCTAGTAGGAT |
|  |  | Reverse | GGTAGTGCTCAGAGGGACAC |
|  | 3‘ UTR | Forward | ACCTCAGAGCTGTACCCCAC |
|  |  | Reverse | GAGCAAAGTCTGGGGGTCAA |
|  | 3‘ UTR | Forward | GTGTCTGTGCTCCTCCTTCC |
|  |  | Reverse | GCTCAAATGCAAGATCGGGG |
|  | 3‘ UTR | Forward | TCTCAGGTTCCTTCGCTGGT |
|  |  | Reverse | ACAGCGCTGCTCTAAAAAGAT |
