# Supplementary material for: Whole exome sequencing of microdissected splenic marginal zone lymphoma: a study to discover novel tumor-specific mutations
Source: BMC Cancer. 2015 Oct 24;15:773. doi: 10.1186/s12885-015-1766-z (PMC4619476; doi:10.1186/s12885-015-1766-z)
Supplement: Additional file 6: Table S6. — Coverage statistics for genes of interest. (DOC 30 kb) [file 12885_2015_1766_MOESM6_ESM.doc]

**Table S5 Coverage statistics for genes of interest**

| Gene | Total reads | Mean coverage | Min | Max |
| --- | --- | --- | --- | --- |
| NOTCH2 | 474815 | 66.9 | 35 | 151.5 |
| MYD88 | 47358 | 49.8 | 1 | 71.5 |
| SMYD1 | 77176 | 52.5 | 15.5 | 163 |
| ZNF608 | 325223 | 71.9 | 5.5 | 201 |
| PDE10A | 159785 | 68.2 | 25.5 | 125.5 |
| KLF2 | 4196 | 12.6 | 1 | 43.5 |
