# Supplementary material for: Whole exome sequencing of microdissected splenic marginal zone lymphoma: a study to discover novel tumor-specific mutations
Source: BMC Cancer. 2015 Oct 24;15:773. doi: 10.1186/s12885-015-1766-z (PMC4619476; doi:10.1186/s12885-015-1766-z)
Supplement: Additional file 7: Figure S1. — Pyrosequencing of validation cohort. Five genes with SNVs in the discovery cohort were analysed for recurrent mutations in a validation cohort (n = 24). Only the SNV position of the respective discovery case was analysed. A. MYD88 794 T > C. B. NOCTH2 7090C > T. C. SMYD1 836G > T. D. PDE10A 1072G > A. E. ZNF608 3659A > G. (DOC 291 kb) [file 12885_2015_1766_MOESM7_ESM.doc]

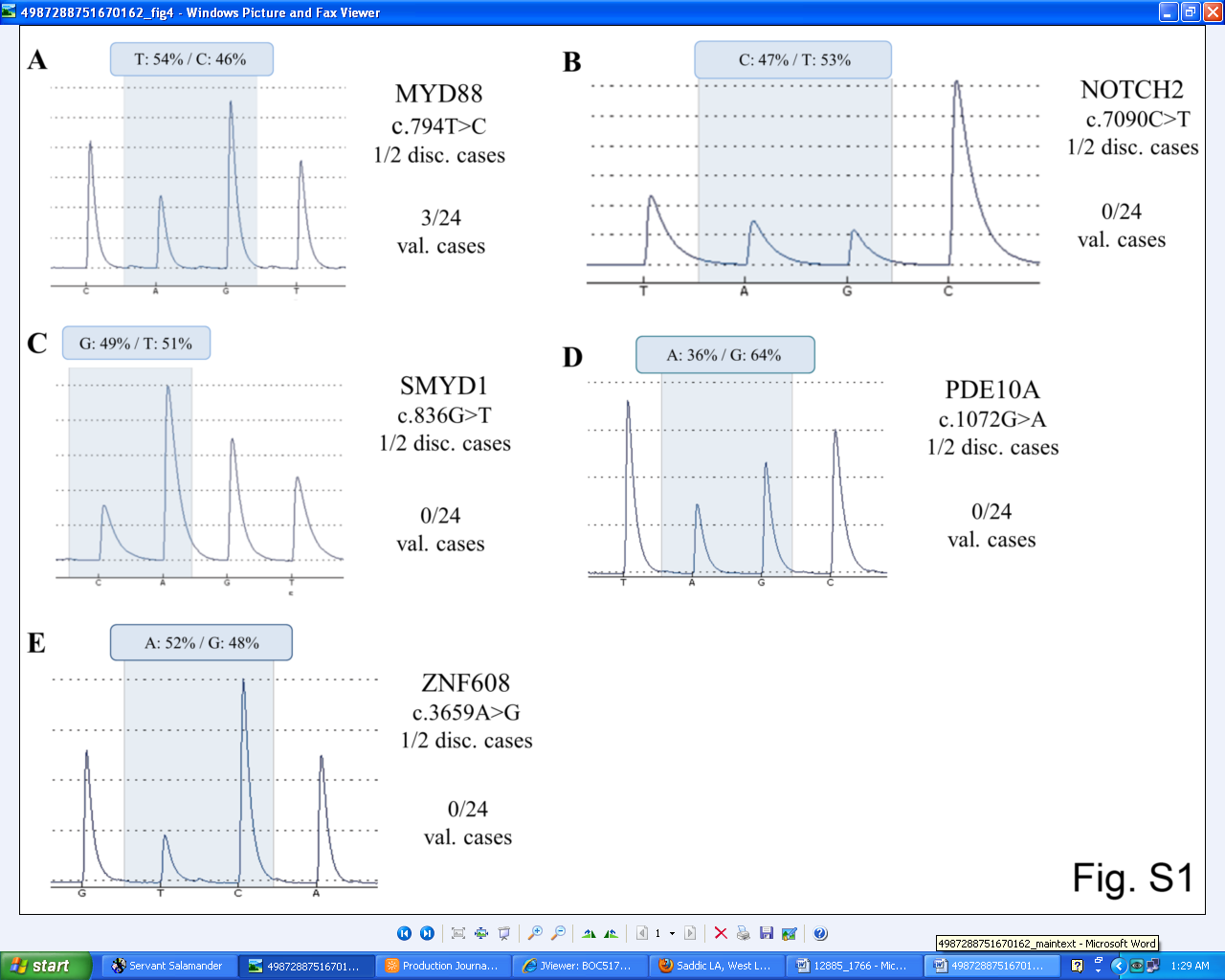


**Figure S1 Pyrosequencing of validation cohort.** Five genes with SNVs in the discovery cohort were analysed for recurrent mutations in a validation cohort (n = 24). Only the SNV position of the respective discovery case was analysed. A. *MYD88* 794 T > C. B. *NOCTH2* 7090C > T. C. *SMYD1* 836G > T. D. *PDE10A* 1072G > A. E. *ZNF608* 3659A > G
