# Supplementary material for: Whole exome sequencing of microdissected splenic marginal zone lymphoma: a study to discover novel tumor-specific mutations
Source: BMC Cancer. 2015 Oct 24;15:773. doi: 10.1186/s12885-015-1766-z (PMC4619476; doi:10.1186/s12885-015-1766-z)
Supplement: Additional file 8: Figure S2. — Sanger validation of NOTCH2 p.Q2364* nonsense mutation. Nucleotide substitution c.7090C > T in the NOTCH2 PEST domain within exon 34. (DOC 263 kb) [file 12885_2015_1766_MOESM8_ESM.doc]

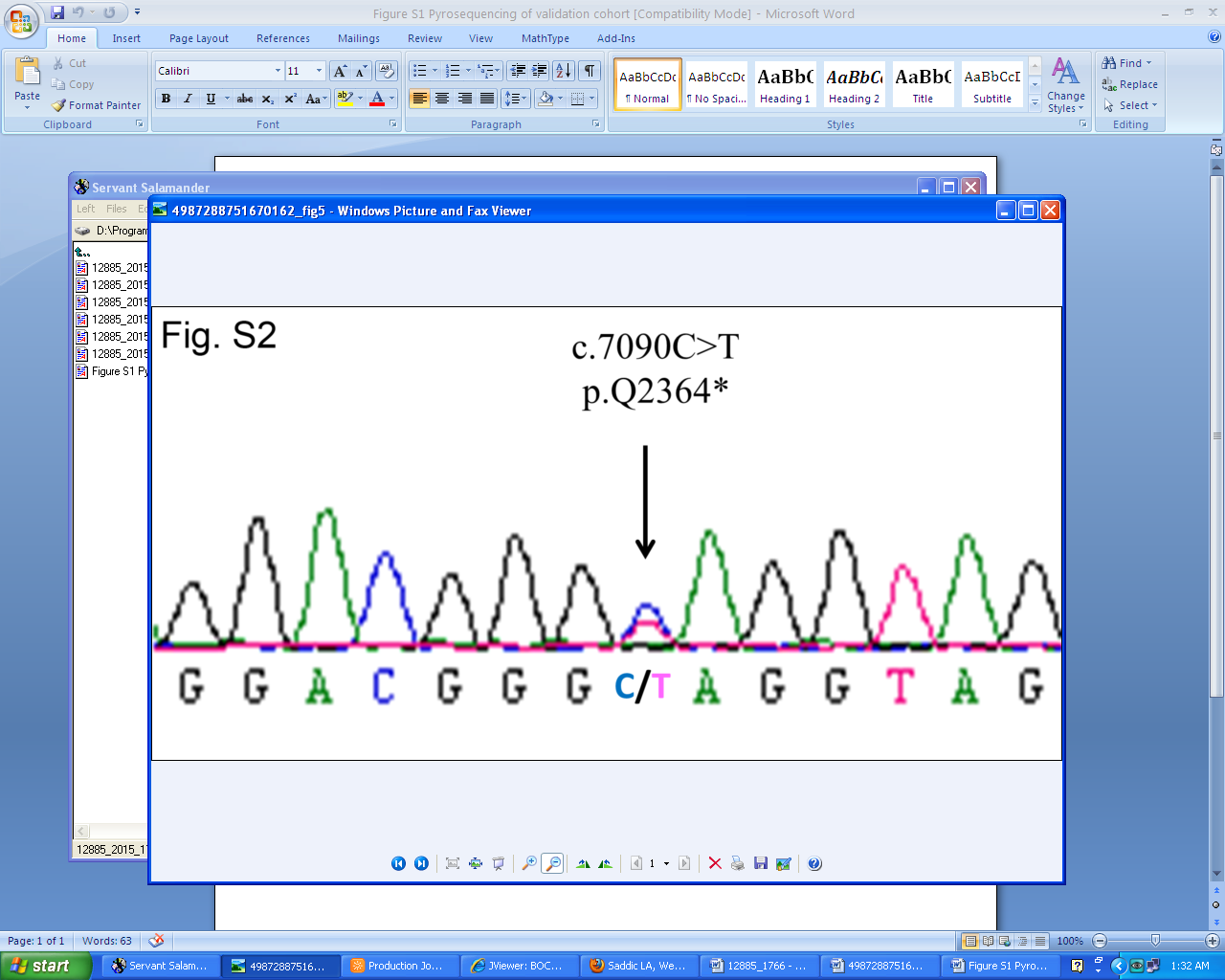


**Figure S2 Sanger validation of *NOTCH2* p.Q2364* nonsense mutation.** Nucleotide substitution c.7090C > T in the *NOTCH2* PEST domain within exon 34.
