# Supplementary material for: Whole exome sequencing of microdissected splenic marginal zone lymphoma: a study to discover novel tumor-specific mutations
Source: BMC Cancer. 2015 Oct 24;15:773. doi: 10.1186/s12885-015-1766-z (PMC4619476; doi:10.1186/s12885-015-1766-z)
Supplement: Additional file 9: Figure S3. — Crystalline structure of the murine SMYD1. Coding mutations found in SMZL are highlighted (c.836G > T; p.C279F in blue, c.962C > A; p.S321Y in red). (DOC 232 kb) [file 12885_2015_1766_MOESM9_ESM.doc]

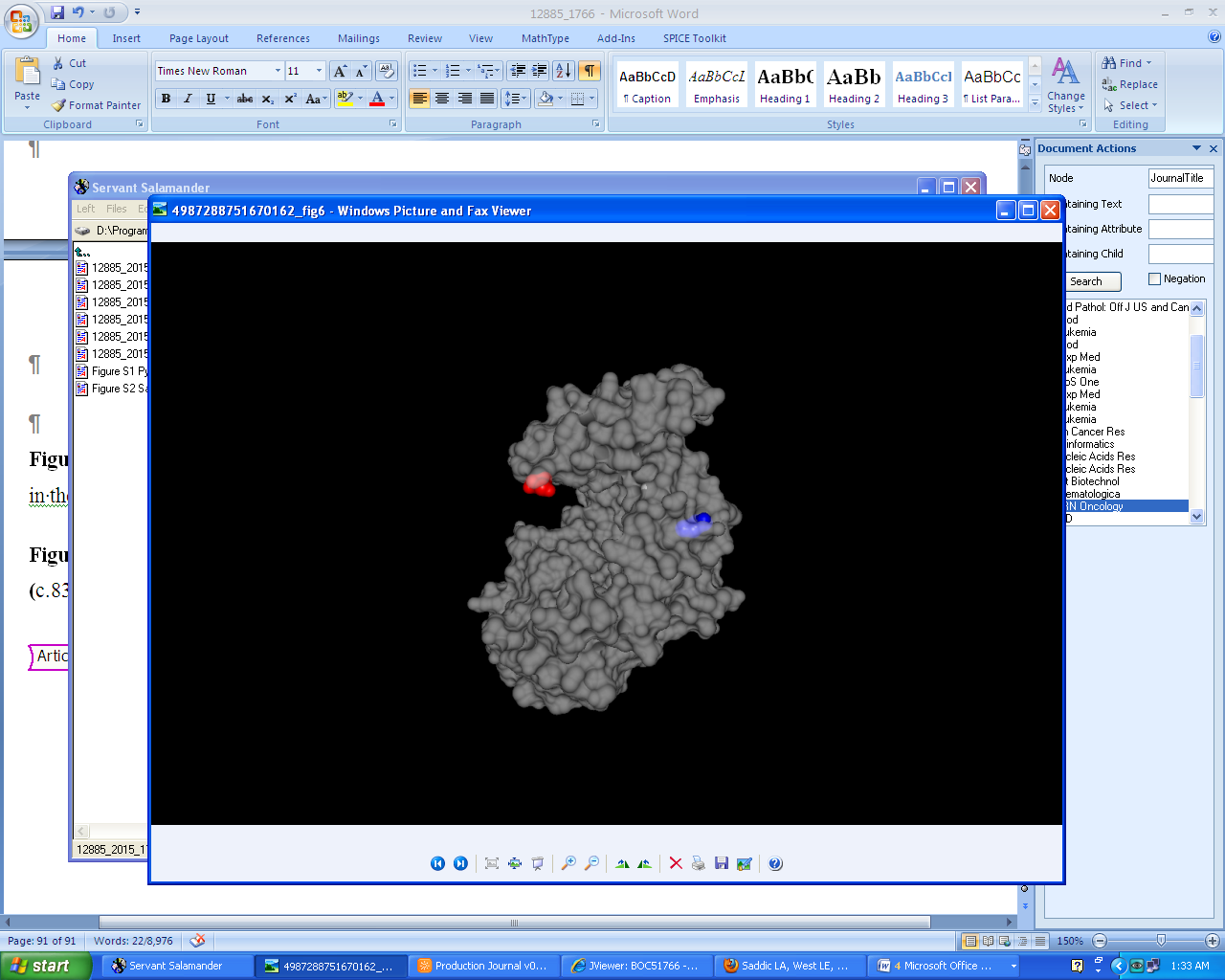


**Figure S3.Crystalline structure of the murine SMYD1**. Coding mutations found in SMZL are highlighted **(**c.836G > T; p.C279F in blue, c.962C > A; p.S321Y in red).
